# ALS Diagnostic Delays in Hispanic/Latinx Patients

**DOI:** 10.1002/alz.091484

**Published:** 2025-01-09

**Authors:** Gabrielle Hromas, Chen‐Pin Wang, Roman A. Fernandez, Carlayne Jackson, A. Campbell Sullivan

**Affiliations:** ^1^ University of Texas Health Science Center at San Antonio, San Antonio, TX USA; ^2^ Glenn Biggs Institute for Alzheimer’s & Neurodegenerative Diseases, University of Texas Health Science Center, San Antonio, TX USA; ^3^ South Texas Veterans Health Care System, Geriatrics Research, Education & Clinical Center (GRECC), San Antonio, TX USA; ^4^ Glenn Biggs Institute for Alzheimer’s & Neurodegenerative Diseases, San Antonio, TX USA; ^5^ University of Texas Health San Antonio, San Antonio, TX USA; ^6^ Division of Neuropsychology, University of Texas Health Science Center at San Antonio, San Antonio, TX USA; ^7^ Glenn Biggs Institute for Alzheimer’s and Neurodegenerative Diseases, University of Texas Health San Antonio, San Antonio, TX USA

## Abstract

**Background:**

Prior research identified delays in diagnosis of Amyotrophic Lateral Sclerosis (ALS) amongst ethnic and racial minorities. This study examined differences in diagnostic delays and symptom severity between White non‐Hispanic and Hispanic/Latinx patients.

**Method:**

To increase the number of Hispanic/Latinx cases, data from the Pooled Resource Open‐Access ALS Clinical Trials (PRO‐ACT), a database including clinical trial participants from nine contributing entities, were combined with a sample of patients recruited from the multidisciplinary ALS clinic at UT Health San Antonio. Two sample T‐test and multivariable regression adjusted for inverse propensity scores weights of ethnic group were conducted to compare ethnic difference in the length of diagnostic delay as well as scores on the ALS Functional Rating Scale‐Revised (ALSFRS‐R; higher scores = less severe symptoms) at the time of first study/medical appointment.

**Result:**

1,916 patients (165 Hispanic/Latinx) with ALS were included in the final sample. Mean ALSFRS‐R scores: 37.8 (5.4) for White non‐Hispanic, 34.2 (7.0) for Hispanic/Latinx. Mean length of diagnostic delay: 390 (392) for White non‐Hispanic, 658 (995) for Hispanic/Latinx. Linear regression showed that White non‐Hispanic patients had shorter delays in diagnosis (p<0.001) and higher (less severe) scores on the ALSFRS‐R (p<0.001).

**Conclusion:**

These results support prior research showing diagnostic delays in non‐White patients with ALS. Results also show that Hispanic/Latinx patients had more severe symptoms at the time of diagnosis than White non‐Hispanic patients. Diagnostic delays slow access to pharmacological interventions and supportive services. These results highlight the need to improve access to medical care across different cultures and ethnicities in the United States.

